# Water–Heat Dual-Activated Scalable Cellulose Film Adhesive

**DOI:** 10.34133/research.1393

**Published:** 2026-08-05

**Authors:** Xia Sun, Zhengyang Yu, Shibo Wang, Jiaying Zhu, Yimin Mao, Dingyuan Zheng, Wenshuai Chen, Feng Jiang

**Affiliations:** ^1^ Sustainable Functional Biomaterials Laboratory, Bioproducts Institute, Faculty of Forestry and Environmental Stewardship, University of British Columbia, Vancouver, BC V6T 1Z4, Canada.; ^2^ Chemical & Biomedical Engineering Department, FAMU-FSU College of Engineering, Florida State University, Tallahassee, FL 32310, USA.; ^3^ Key Laboratory of Bio-based Material Science and Technology of Ministry of Education, Northeast Forestry University, Harbin, China.

## Abstract

Despite their widespread use, synthetic adhesives raise substantial concerns regarding environmental impact, toxicity, recyclability, and volatile organic compound emissions, while their predominantly liquid nature often requires energy-intensive curing processes. Here, we report an all-cellulose film adhesive derived from renewable cellulose through a simple oxidation–reduction modification that transforms cellulose into a linear heat-softenable structure. The resulting material can be activated by either water or heat to function as an effective wood adhesive. Upon water activation, the cellulose film bonds thick wood panels into structural composites without external heating, achieving lap shear and compressive shear strengths of 3.1 and 6.5 MPa, respectively. This room-temperature, water-triggered adhesion reduces processing energy demand and is well suited for manufacturing engineered wood products. Owing to its thermoplastic behavior, the adhesive also enables heat-activated bonding of wood and paper, including thermal sealing of paper bags. This work establishes a renewable and low-emission adhesive platform, advancing sustainable alternatives to conventional petrochemical adhesives.

## Introduction

Adhesives, which play an essential role across domestic and industrial applications, are predominantly petroleum-derived, including polyacrylics, ethylene-vinyl acetate, epoxy, formaldehyde-based resin, and polyurethane [1,2]. Beyond environmental concerns, industrial adhesive production commonly uses toxic chemicals such as isocyanate and formaldehyde, posing risks to human health [3]. Among all types of adhesives, wood adhesives [4,5] have recently garnered marked attention due to their potential to support green and sustainable product development. With a well-established market, wood adhesives are projected to reach a market size of USD 6.34 billion by 2025 [6] primarily driven by the growing demand for wood products. Despite its growth, the wood adhesive industry faces several challenges, including health and environmental concerns, such as the emission of carcinogenic formaldehyde and the nonrecyclability and nondegradability of the heavily crosslinked structures. In fact, the latter issue has hindered the wood recycling process, resulting in large amounts of construction and demolition waste accumulating in landfills without a second life [7,8]. For example, the removal of urea-formaldehyde (UF) resin typically requires an additional heating process combined with acid treatment [9,10], which can lead to concomitant degradation of the wood fibers. Besides, the residual UF adhesives can reduce the shear strength of recycled wood panels by up to 50% [11]. Therefore, developing non-petroleum-based adhesives that exhibit low toxicity, high bonding strength, and good recyclability is imperative [6,12,13].

Recently, biobased adhesives have attracted broad attention in the wood industry, with examples including those derived from lignin [5], carbohydrates [14–16], tannin [17], and proteins [18]. For example, Shuai and colleagues [5] prepared lignin-based wood adhesive from lignocellulosic biomass by separating uncondensed or slightly condensed lignin from biomass. The aqueous lignin suspension can be directly applied as an adhesive on wood veneers with a maximum adhesion shear strength of ~1.3 MPa. Besides, dialdehyde starch adhesives [4,19], synthesized by ring-opening oxidation, have been commercialized for fabricating fiberboards and particleboard [20,21]. In addition, we have recently developed an all-cellulose hydrogel adhesive composed of concentrated dialcohol nanocellulose [14]. However, these adhesives usually exist in liquid form such as water-based emulsions [22], hydrogels [14], and organic solvent-based solutions [23]. The liquid state presents drawbacks, including emission of volatile organic compounds (VOCs), relatively short shelf-life, high transportation costs, and the need for excessive energy during curing. Therefore, developing solid-state adhesives that can be readily activated upon application is of great interest for both environmental sustainability and energy efficiency.

Despite being the most abundant polymer on Earth [24–26], cellulose has not been widely studied for adhesive applications, primarily due to its rigid anhydroglucose ring structures and dense inter- and intra-molecular hydrogen bonding, which limit its ability to interact and bond with adherends [14,27–30]. Herein, we propose the synthesis of an all-cellulose solid film adhesive that can be activated by either water or heat. Rigid cellulose anhydroglucose rings are converted into flexible linear chain structures with enhanced chain mobility through the sequential periodate oxidation and sodium borohydride reduction of pulp fibers. The synthesized cellulose suspension can be facilely cast into a freestanding and dry film, showing strong adhesion toward various substrates. For example, the all-cellulose film adhesive exhibits high shear strength toward wood (3.08 ± 0.14 MPa activated by water, and 2.23 ± 0.16 MPa activated by heat). Additionally, large-scale film adhesive can be fabricated without volatile organic solvents or formaldehyde-containing components, offering a scalable and industrially relevant pathway toward a fully bio-based adhesive. By integrating the functionalities of water-activated and heat-softening adhesives within a single cellulose film, this work introduces a new paradigm in sustainable adhesive design.

## Results and Discussion

### Design concept of all-cellulose film adhesive

**Fig. 1.**
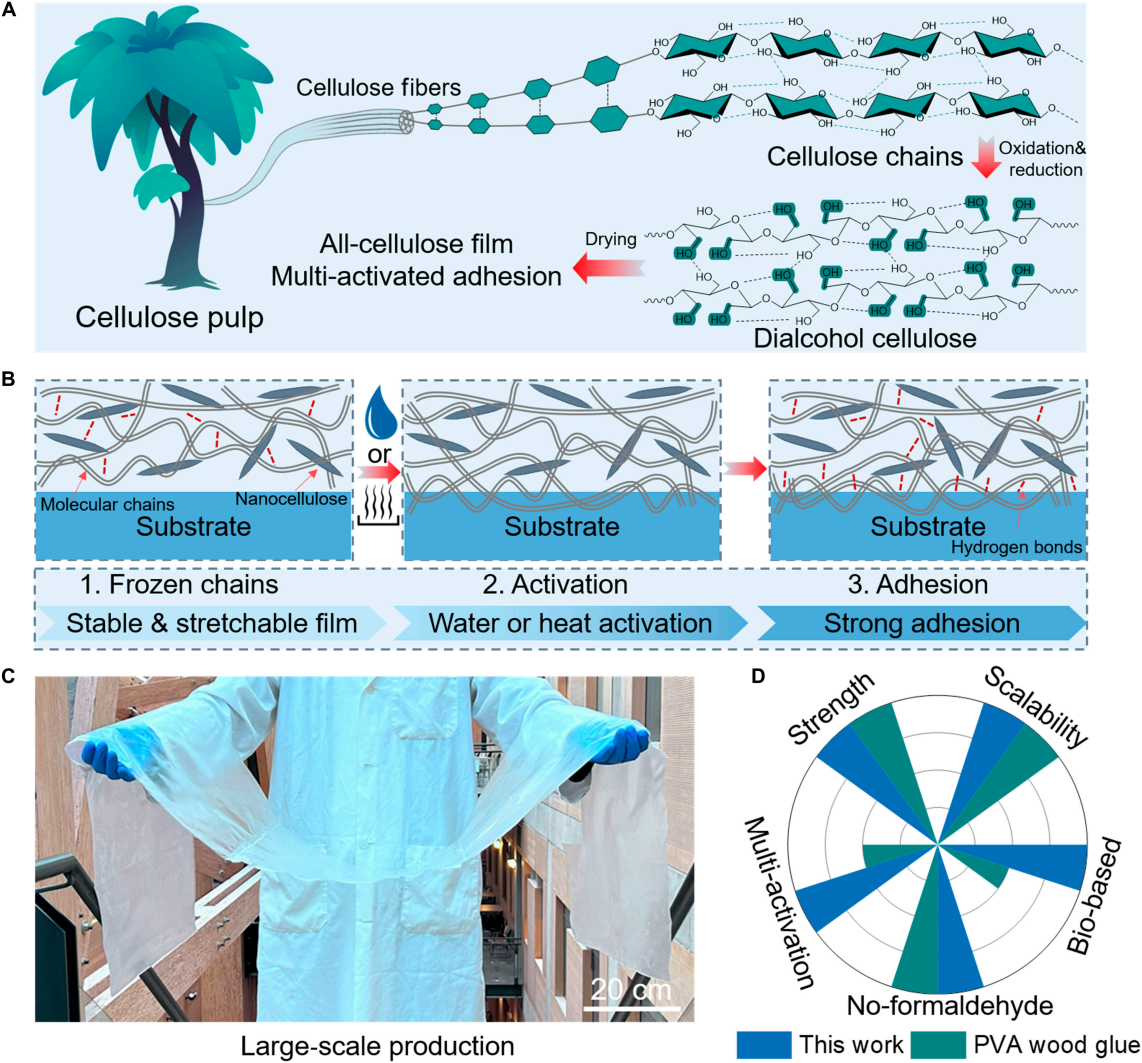
Design concept of the all-cellulose film adhesive. (A) Schematic illustration of the preparation of dialcohol cellulose from pulp fibers through oxidation and reduction. (B) Schematical illustrations of film adhesion activation process. The cellulose chains within the dry film are locked (left). After being activated by heat or water, the cellulose chains gain enhanced mobility and can penetrate substrates to form stable mechanical interlocking (middle). A dense hydrogen bonding network, along with mechanical interlocking, forms between the cellulose film and substrate upon the removal of activators (right). (C) Photo of a large piece of dialcohol cellulose film (length: 200 cm; width: 40 cm). (D) Radar chart showing the comparison between the commercial PVA wood adhesive and cellulose film adhesive.

As described previously, the lack of adhesion property for cellulose is primarily attributed to its rigid anhydroglucose rings and the strong inter- and intra-molecular hydrogen bonds, both of which constrain molecular mobility and limit interactions with adherends. To enhance chain mobility and adhesion, the rigid anhydroglucose rings were cleaved by sequential oxidation and reduction. As illustrated in Fig. 1A, selective periodate oxidation cleaves the C2–C3 bond of anhydroglucose units (AGUs) and generates dialdehyde cellulose, followed by borohydride reduction of the aldehyde groups into primary hydroxyl groups (Fig. S1). Therefore, the resulting dialcohol cellulose consists of unmodified anhydroglucose units and C2–C3 ring-opened dialcohol groups along the molecular chain [30–33]. This oxidation transformed the rigid ring into a linear form, while the reduction increased the number of primary hydroxyl groups, thereby strengthening hydrogen bonding with the substrate [14]. Due to the enhanced hydrophilicity and heat-softenable behavior, the dialcohol cellulose film can be easily activated by water or heat, serving as adhesives to bind diverse substrates. Figure 1B illustrates the activation process of the cellulose film adhesive. In the dry state, the cellulose chains and nanocellulose are locked with limited mobility. However, their mobility can be enhanced by adding water or heating the film above its glass transition temperature, allowing for the molecular chains to reorganize and penetrate the substrate. Through this reorganization, the dialcohol cellulose establishes strong cohesive interactions with itself as well as adhesive bonds with the adherend. Once the activators (water or heat) are removed, the cellulose chains stabilize, forming a robust adhesive layer.

Due to the excellent film-forming properties of the dialcohol cellulose, it demonstrates good scalability and can be readily processed into large films (Fig. 1C). Compared with a commercial polyvinyl acetate (PVA) wood adhesive (Fig. 1D and Fig. S2), the cellulose film adhesive developed here offers a fully bio-based composition, solid-state film format, and dual water/heat activation capability while maintaining practical bonding performance. Besides, we anticipate that the strategy of hydrogen bonding regulation through sequential oxidation and reduction will also provide a foundation for designing strong adhesives from other polysaccharides.

### Structural evolution by sequential oxidation and reduction

**Fig. 2.**
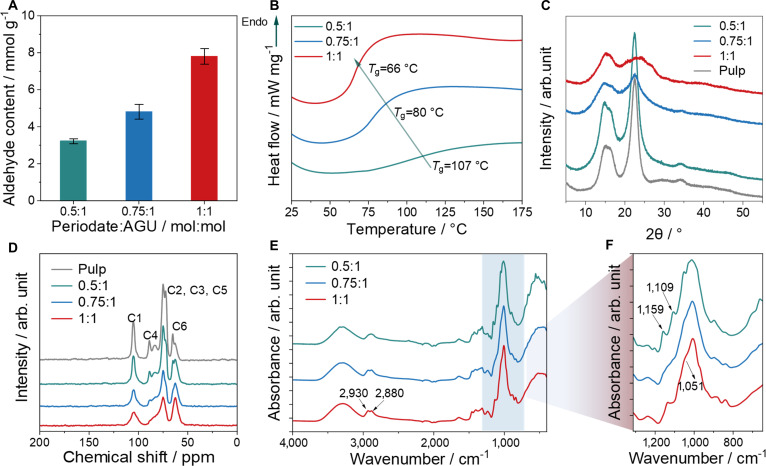
Structural evolution of dialcohol celluloses with different oxidation degrees. (A) Aldehyde contents. (B) DSC curves. (C) XRD diffractogram. (D) ^13^C CP-MAS NMR spectra. (E) ATR-FTIR spectra. (F) Enlarged spectra.

The effects of oxidation–reduction on morphology, chain mobility, crystallinity, and primary hydroxyl group content of dialcohol cellulose were investigated by varying the periodate-to-AGU molar ratio, with the hypothesis that these structural changes ultimately influence adhesion performance. Optical microscopy (OM) with and without cross-polarizer was employed to observe the microstructures of kraft pulp after oxidation and reduction. As shown in Figs. S3 to S5, when increasing the periodate-to-AGU molar ratio from 0.5:1 to 0.75:1, a substantial reduction in the fiber length can be observed, indicating the chain scission by the periodate oxidation [34]. No obvious microfibers are observed with further increasing the periodate-to-AGU ratio to 1:1, which can also be reflected by the high transparency of the 1:1 suspension (Fig. S6). This suggests that increasing the periodate enhances the degree of oxidation, leading to the fragmentation of kraft pulp into smaller pieces. As the cellulose suspension became increasingly transparent with higher periodate dosages, it is hypothesized that some of the cellulose was converted into nanocellulose, which is invisible under an optical microscope. Therefore, transmission electron microscopy (TEM) was employed to observe the nanostructures as shown in Fig. S7. With increasing periodate-to-AGU ratio, the dialcohol cellulose transformed from long nanofiber (0.5:1) to short nanorod (1:1). This indicates that with increasing periodate-to-AGU ratio, more cellulose fibers have been attacked and cleaved, and thus form more nanocellulose [34]. The aldehyde contents (Fig. 2A) in the oxidized cellulose with periodate-to-AGU ratios of 0.5:1, 0.75:1, and 1:1 were measured using hydroxylamine hydrochloride titration [34], which were determined as 3.92, 5.63, and 8.72 mmol/g, corresponding to the degree of oxidation of 31.7, 45.6, and 70.7%, respectively. These aldehyde groups were further reduced to primary hydroxyls by borohydride reduction for further characterization and applications.

Structural alterations in a polymer can affect its response to heat. This was evaluated using differential scanning calorimetry (DSC) to characterize the dialcohol cellulose with different oxidation degrees (Fig. 2B). In the temperature range from 25 to 175 °C, all 3 dialcohol celluloses showed sudden upward shifts, indicating the existence of glass transition temperature (*T*_g_). With the increasing oxidation degree, the *T*_g_ shifted from 107 °C (0.5:1) to 66 °C (1:1). The shift in *T*_g_ manifested that the ring-opening reaction greatly increased chain mobility. This enhanced chain mobility also aligns with the x-ray diffraction (XRD) diffractogram displayed in Fig. 2C, which indicates a gradual decrease in crystallinity and a transition toward a more amorphous structure. This oxidation-dependent hierarchy defines the balance between flexibility and adhesion strength observed in the films. ^13^C cross-polarization magic-angle spinning nuclear magnetic resonance (CP-MAS NMR) was further employed to characterize the structural evolution of dialcohol celluloses. As shown in Fig. 2D, after oxidation and reduction, the C4 peak in the original pulp gradually decreased, indicating a lower crystallinity with a higher oxidation degree. The signals in the 55- to 65-ppm (parts per million) region gradually increased, while the 70- to 80-ppm region gradually decreased, implying the shift of secondary hydroxyl groups to primary hydroxyl groups. Attenuated total reflectance (ATR)-Fourier transform infrared (FTIR) spectroscopy was further utilized to study the changes in typical functional groups. As shown in Fig. 2E, with high oxidation ratios, the absorbance band of -CH_2_- asymmetric stretching at 2,930 cm^−1^ became clearer, implying more -CH_2_OH groups [34]. In addition, as seen from the enlarged spectra (Fig. 2F), the absorbance band assigned to primary -OH groups at 1,051 cm^−1^ increased, while the absorbance band assigned to secondary -OH groups at 1,109 cm^−1^ decreased gradually [31], also implying that more primary hydroxyl groups were formed. Besides, due to the chain scission reaction through hydrolytic scissions and β-alkoxy elimination [34] during oxidation, the absorbance band at 1,159 cm^−1^ assigned to asymmetric stretching of C–O–C bonds in the glycosidic linkage also decreased. These oxidative chain scissions resulted in a modest decrease in mass yield compared to the starting pulp (81.9% at 1:1 oxidation ratio; Fig. S8). All these results suggested that with the increase of periodate-to-AGU ratio, the dialcohol cellulose became more amorphous, exposed more primary hydroxyl groups, and exhibited enhanced chain mobility, which helped to regulate the hydrogen bonding formation and the adhesion performance.

### Properties and structures of all-cellulose film adhesive

**Fig. 3.**
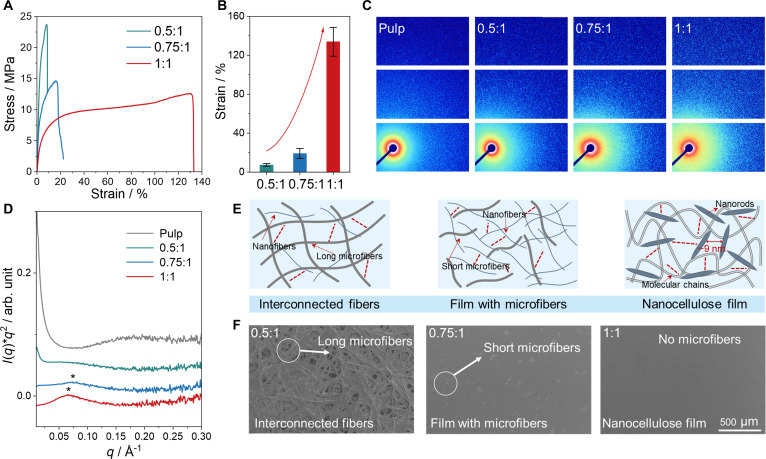
Mechanical properties and structures of dialcohol cellulose films with different oxidation degrees. (A) Typical stress–strain curves and the corresponding (B) tensile strain under different oxidation degrees. (C) 2D SAXS images of pulp and dialcohol cellulose films with different oxidation degrees. (D) The Lorentz-corrected scattering intensity *Iq^2^* versus vector *q*. The asterisk represents the peak by Lorentz fitting. Numbers in the legend (e.g., 0.5:1, 0.75:1, 1:1) indicate periodate-to-AGU ratios of the samples. (E) Schematic illustration showing the structures of dialcohol cellulose under varying oxidation degrees. (F) Surface morphologies of all-cellulose films with different oxidation degrees.

As previously discussed, the periodate-to-AGU ratios influence the morphology and chain mobility of dialcohol cellulose, leading to morphological variance of the corresponding films. At a lower oxidation degree, the film exhibited a porous structure with visible microfibers. In contrast, much smoother film morphologies were observed at higher oxidation levels, particularly at the 1:1 ratio (Figs. S9 and S10). As a result of these morphological differences, an increase in oxidation degree results in films with greater transparency (Figs. S11 and S12), attributed to the generation of more nanoscale cellulose at higher periodate-to-AGU ratios. Figure 3A displays the typical stress–strain curves of dialcohol cellulose film with different oxidation degrees, revealing the mechanical properties of films. The tensile strain substantially increased from 7.3% (0.5:1), 19.2% (0.75:1) to 134.0% (1:1), with corresponding enhanced toughness (1.33 ± 0.18, 3.03 ± 0.68, and 13.87 ±1.43 MJ/m^3^, respectively) shown in Fig. 3B and C. Sequential oxidation and reduction convert part of the rigid anhydroglucose rings in cellulose into ring-opened linear structures with increased primary hydroxyl groups. Although these additional hydroxyl groups enhance the potential for intermolecular hydrogen bonding, the cleavage of the C2–C3 covalent bond disrupts the original ring-constrained structure and increases segmental mobility, consistent with the decreased *T*_g_ and reduced crystallinity [14,33]. Therefore, the newly formed hydrogen bonds mainly act as dynamic and reversible sacrificial interactions that can dissociate and reform during deformation, promoting energy dissipation and improved toughness. In contrast, the disrupted pyranose-ring structure and reduced crystallinity lead to a corresponding decrease in tensile strength and modulus (Fig. S13A and B).

To further investigate the nanostructures of the dialcohol cellulose films, small-angle x-ray scattering (SAXS) was employed. The 2-dimensional (2D) SAXS images (Fig. 3C) revealed that all the dialcohol cellulose films represented an isotropic structure regardless of their oxidation degrees. As depicted in Fig. 3D, the characteristic internal spacing was estimated from the peak position of the Lorentz-corrected SAXS profiles. Within the observed *q* range, the pulp displayed a broad peak at approximately 0.18 Å^−1^, corresponding to a spacing of ~3.5 nm, which can be assigned to the characteristic distance between elementary fibrillar domains. With increasing oxidation degree, the scattering feature shifted to lower *q*, indicating an increased characteristic spacing, reaching ~9.2 nm for the 1:1 sample. The 1:1 sample also exhibited a slightly sharper correlation peak, reflecting a narrower distribution of inter-domain spacing. This indicates that the highly oxidized cellulose contains shorter and more uniformly distributed nanoscale domains, consistent with the TEM observations (Fig. S7). Based on these findings, the film structures were schematically depicted in Fig. 3E. As the oxidation degrees increased, the nanosized celluloses became shorter with a more separated structure, likely filled with amorphous chains. Scanning electron microscopy (SEM) results further supported the hypothesis. As shown in Fig. 3F, at a lower oxidation degree, the film exhibited a porous structure with visible microfibers. In contrast, much smoother film morphologies were observed at higher oxidation levels, particularly at the 1:1 ratio. Consistent with other results, the separated dialcohol nanocelluloses and the flexible amorphous structures accounted for the energy dissipation during the external deformation process and therefore inducing the ultra-stretchability of dialcohol cellulose film (134.0% strain, 1:1 group).

### Water and heat activated adhesion performance

**Fig. 4.**
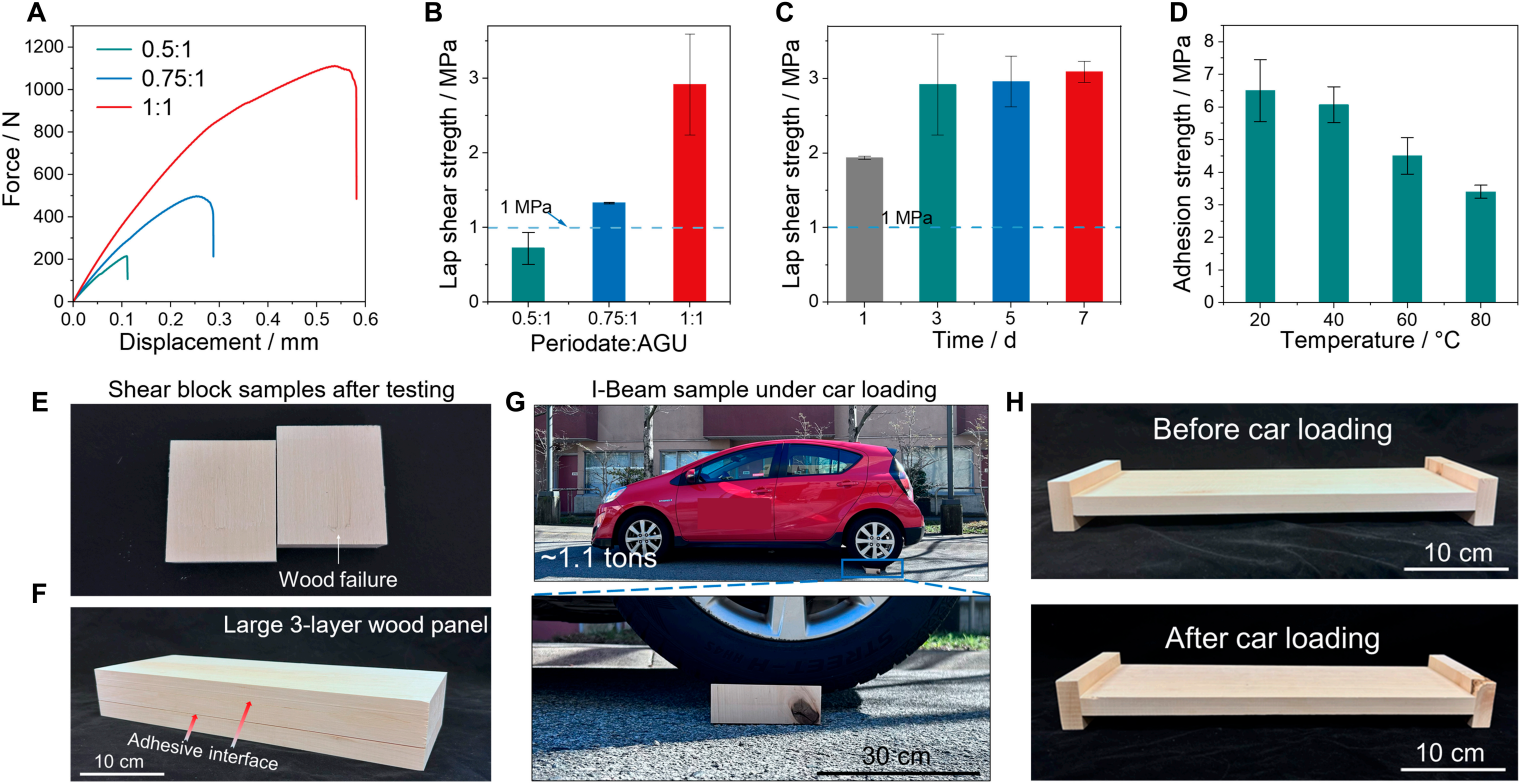
Water-activated adhesive performances and demonstration of applications. (A) Force–displacement curves of dialcohol celluloses film adhesives with different oxidation degrees and (B) corresponding lap shear strength. (C) Lap shear strength under different curing times. (D) Shear block strength of 1:1 film under different temperatures. (E) Digital photos of shear block samples after testing showing typical wood failure. (F) Three-layer wood panel (length: 40 cm; width: 15 cm) bonded by 1:1 film adhesive. (G) Photo of an I-beam structure bonded with the film adhesive, supporting a 1.1-ton vehicle. (H) I-beam before and after car loading testing, maintaining structural integrity.

Due to the enhanced chain mobility and hydrophilicity imparted by the ring-opened dialcohol structure and abundant primary hydroxyl groups, the film adhesive can be activated by water or heat to achieve effective adhesion on basswood. The flexible dialcohol segments are expected to promote interfacial hydrogen bonding with wood, while the remaining rigid cellulose/nanocellulose domains may contribute to cohesive reinforcement within the adhesive layer. Lap shear tests (Fig. S14) were first employed to evaluate the water-activated adhesion properties. After applying the dialcohol cellulose films on wood substrates, followed by water spraying and room temperature curing, the dialcohol cellulose film can firmly bond to the wood substrates through hydrogen bonds and mechanical interlocking. As shown in Fig. 4A and B, the force–displacement curves (solid content: ~6 mg/cm^2^; Fig. S15) demonstrated that as the oxidation degree increased, the lap shear strength improved from 0.72 ± 0.21 MPa (0.5:1), 1.33 ± 0.01 MPa (0.75:1) to 2.91 ± 0.67 MPa (1:1). Curing time was another important factor that affected the adhesion strength. As shown in Fig. 4C and Fig. S16, extending the curing time from 1 to 7 d initially increases lap shear strength from 1.91 ± 0.02 MPa (1 day) to 2.91 ± 0.67 MPa (3 d), eventually plateauing at 2.96 ± 0.34 MPa (5 d) and 3.08 ± 0.14 MPa (7 d). The curing time can be potentially further reduced by moderate heating, as indicated by following heat activation experiments. This strength meets the requirement of 1 MPa (ISO Standard 12466-2: 2007) and is comparable to most reported wood adhesives, as shown in Table S1 [5,35–41]. A simple demonstration (Fig. S17) highlighted the high bonding strength of the all-cellulose film adhesive, where 2 pieces of wood bonded by a 1:1 film adhesive with an overlapped area of ~4 cm^2^ could withstand the weight of an adult (~75 kg) without breaking. During curing, water can facilitate the penetration of cellulose chains into wood substrates due to the increased chain mobility in water. Therefore, high adhesion strength can be achieved through the synergy of hydrogen bonding, mechanical interlocking, and cohesive reinforcement. Water plasticization and heat activation increase the mobility of the ring-opened dialcohol cellulose chains, enabling better conformal contact with the porous wood surface. The flexible dialcohol segments promote interfacial hydrogen bonding with wood, while the remaining rigid cellulose and nanocellulose domains reinforce the adhesive layer. This mechanism is supported by the clear wood failure after lap shear and shear block tests (Fig. 4E and Figs. S18 to S21) and by the lower adhesion strength observed on smooth nonporous substrates, where mechanical interlocking is limited. Also, shear block tests were employed to evaluate the adhesion strength of the 1:1 film adhesive. As shown in Fig. 4D and Fig. S22, at room temperature (20 °C), the wood block samples exhibited a high compressive shear adhesion strength of 6.5 ± 0.9 MPa, with clear wood failure (Fig. 4E). While the adhesion strength decreased with rising temperatures due to the heat-softenable nature of the dialcohol cellulose, it still maintained a practical adhesion strength of 3.4 ± 0.2 MPa at 80 °C, demonstrating its potential application under a moderately elevated temperature environment.

Large cellulose film adhesives can be readily fabricated using a large mold (Fig. 1C), and their ability to cure at room temperature via water activation makes them suitable for bonding dimensional lumber. A large 3-layer wood panel (basswood, adhesive solid content: ~6 mg/cm^2^; thickness: 2 cm, glued area: ~40 × 15 cm^2^) was prepared, as shown in Fig. 4F, revealing the potential of the film adhesive for constructing wood composites using dimensional lumber. Furthermore, an I-beam (solid content: 5.2 mg/cm^2^; glued area: ~50 cm^2^) was constructed. As displayed in Fig. 4G and H, the prepared I-beam successfully withstood the weight of a car (1.1 tons) for 10 min. After the car was removed, the I-beam maintained shape integrity and stability, underscoring the high bonding strength and the practical potential of the all-cellulose film adhesives.

**Fig. 5.**
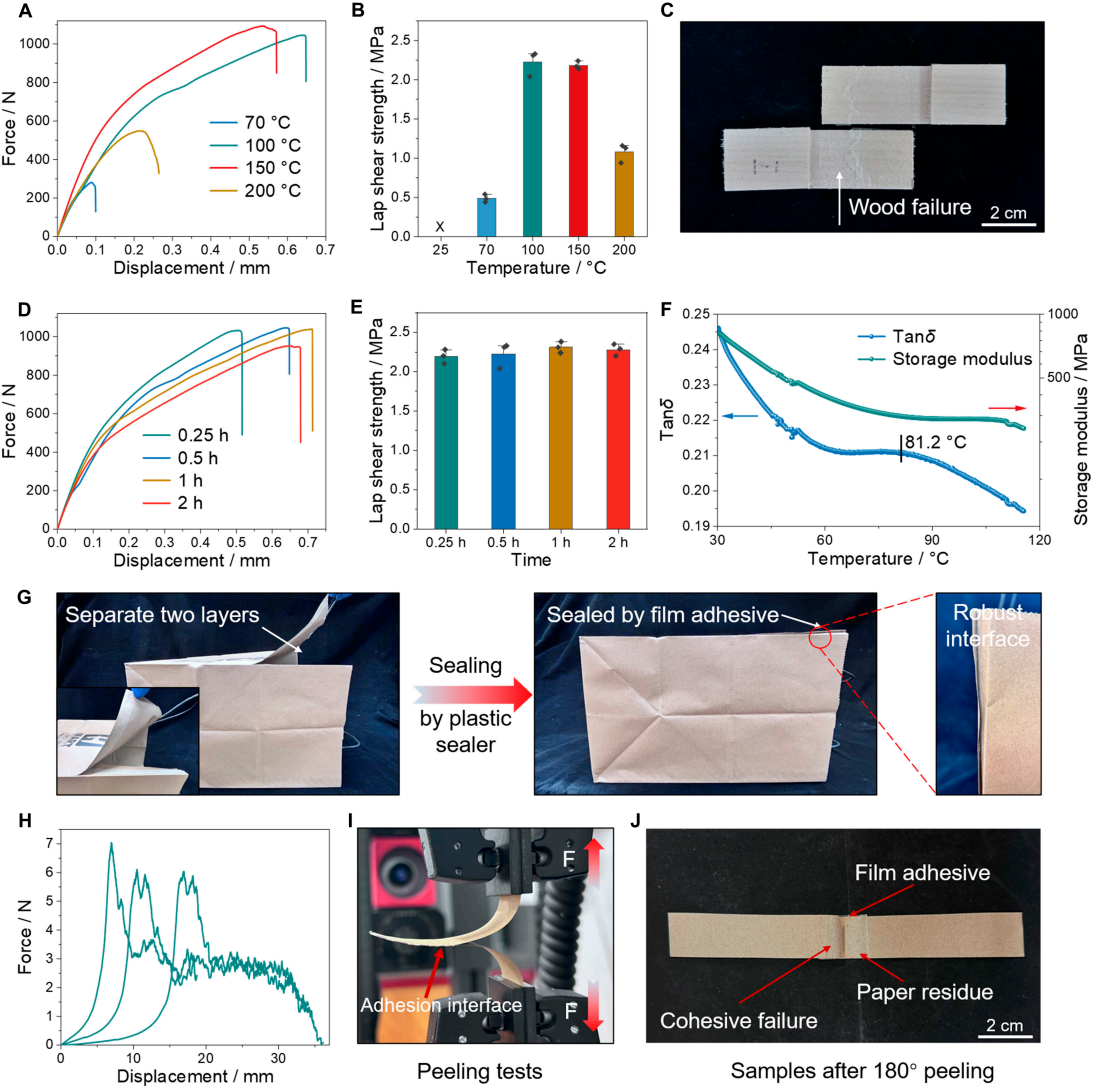
Heat-activated adhesion performances of film adhesives. (A) Force–displacement curves of 1:1 film adhesive activated by heat under different temperatures (time: 0.5 h) and (B) corresponding lap shear strength. (C) Photo of wood failure after adhesion (temperature: 100 °C). (D) Force–displacement curves of the 1:1 film adhesive activated by heat under different activation times and (E) corresponding lap shear strength. (F) Storage modulus and tan δ of the 1:1 film under temperature sweep from 30 to 120 °C (moisture content: 8.2 ± 0.3 wt %) (G) Demonstration of film adhesive by sealing a paper bag through a commercial plastic sealer. Left: Separate 2 layers of paper bag. Middle: Sealed paper bag by a plastic sealer. Right: Enlarged photo of the adhesion interface. (H) Force–displacement curves of 180° peeling tests of 1:1 film adhesive on a paper bag and corresponding photos showing (I) the peeling tests and (J) cohesive failure.

Due to its heat-softenable properties shown in Fig. 2B, the film adhesive can also be activated by heat. As shown in Fig. 5A and B, under low temperatures (e.g., 25 °C), the polymer chains were locked with limited mobility, preventing adhesion to the wood substrate. However, as the temperature exceeds the *T*_g_, the polymer chains exhibit enhanced mobility, enabling them to penetrate the wood substrates and establish strong adhesion (2.23 ± 0.16 MPa at 100 °C), as evidenced by the wood failure observed after testing (Fig. 5C). Nevertheless, further increasing the temperature to 200 °C resulted in decreased adhesion strength (1.08 ± 0.12 MPa), which is likely associated with thermally induced oxidative degradation and/or chain scission of cellulose, as evidenced by the formation of carbonyl groups after heating (Fig. S23). Figure 5D and E demonstrates that the films consistently exhibited high bonding strength (over 2.1 MPa) across pressing times ranging from 0.25 to 2 h, indicating that even short pressing times are sufficient to establish stable adhesion. This fast heat activation route helps address the extended curing time required for water activation and supports the feasibility of the film adhesive for thermal processing. Therefore, the dual-activation capability offers a practical balance between energy efficiency and processing speed. Water activation enables low-energy, ambient curing for applications where extended curing time is acceptable. In contrast, heat activation exploits the film’s heat-softening window to achieve comparable bond strength much faster. Thus, water activation is preferred when heating is impractical, while heat activation is ideal for rapid industrial thermal processes (e.g., hot-sealing).

The high bonding strength observed in heat-activated adhesion can be attributed to 2 key factors: hydrogen bonds and mechanical interlocking, as schematically illustrated in Fig. S24. As previously discussed, a higher oxidation degree leads to the generation of more flexible primary hydroxyl groups and a progressively lower *T*_g_ (Fig. 2B). Consequently, when heat activation occurs above 80 °C, the polymer chains can migrate at the interface, forming stable mechanical interlocks and dense hydrogen bonds with the substrates, resulting in strong bonding strength. Dynamic mechanical analysis (DMA) was employed to support our hypothesis further. As shown in Fig. 5F, the 1:1 film displayed a typical *T*_g_ at 81.2 °C, confirming that the polymer chains displayed increased mobility and flexibility under heat activation. As demonstrated above, the improved flexibility enhances conformability and energy dissipation, thereby improving practical adhesion performance (cohesive failure observed; Fig. 5H to J) and enabling applications such as deformable/packaging seals, conformal lamination, and integration into soft devices. For example, leveraging this heat-activated adhesion capability, the film adhesive can easily seal a paper bag by forming a strong adhesion (Fig. 5G) between 2 paper layers using a commercial plastic sealer. Both lap shear tests (Fig. S25) and 180° peeling tests (Fig. 5H to J) revealed a typical cohesive failure, implying strong adhesion behavior. Beyond wood, the film adhesive can also be applied to other substrates as shown in Fig. S26A and B and Table S2. Compared with porous wood substrates, relatively smooth and nonporous substrates such as glass, metals, and polyethylene terephthalate (PET) exhibited lower adhesion strength, likely due to the reduced mechanical interlocking and weaker interfacial interactions with cellulose chains.

The solid-state format also endowed the cellulose film adhesive with good storage stability. After being stored as a dry film under ambient room conditions for 1 year, the adhesive retained its freestanding film form and could still be activated by heat for wood bonding. The heat-activated lap shear strength of the 1-year-stored film was 2.12 ± 0.46 MPa (Fig. S27), comparable to that of the freshly prepared film adhesive (2.23 ± 0.16 MPa). This result suggests that the cellulose film adhesive can be stored as a dry film at room temperature without special storage conditions, although prolonged exposure to high humidity or direct water contact should be avoided because of its hydrophilic nature.

These results suggest that the present adhesive system is particularly advantageous for porous lignocellulosic materials, while future improvements for non-wood substrates may involve surface treatment or interfacial functionalization to enhance substrate–adhesive interactions. All these results demonstrate that the film adhesive holds great potential for replacing commercial adhesives in packaging applications.

## Conclusion

This work presents a sustainable and versatile strategy for designing biomass-derived adhesives by transforming cellulose into a flexible, high-performance solid film adhesive through sequential oxidation and reduction. The resulting dialcohol cellulose films exhibit tunable chain mobility and abundant hydroxyl groups, enabling strong adhesion to wood through synergistic hydrogen bonding and mechanical interlocking. Compared with previously reported cellulose- or polysaccharide-based adhesives, which are predominantly liquid-based systems, the present work demonstrates a scalable solid-state all-cellulose adhesive capable of both water- and heat-activated bonding within a single material platform. The adhesive achieves comparable strength with other industry-relevant adhesives, demonstrates both scalability and versatility across applications ranging from structural wood composites to flexible packaging. This work provides a general design concept for high-performance polysaccharide materials through controlled hydrogen bonding and chain mobility.

We note, however, that several practical limitations still exist, especially regarding environmental stability under demanding service conditions. Heat activation requires access to heating equipment with precise temperature control, because excessive temperature may enhance chain mobility or induce thermal degradation, leading to reduced adhesion strength. Water activation is energy-efficient but requires longer curing times of several days to reach full strength. In addition, because the present adhesive mainly relies on hydrogen bonding and mechanical interlocking, prolonged exposure to high moisture or water may plasticize the adhesive layer and weaken interfacial hydrogen bonding. Therefore, the current adhesive system is primarily intended for dry or moderate indoor environments rather than prolonged wet or severe outdoor conditions. The long-term stability under ultraviolet (UV) exposure also requires further systematic evaluation. Future improvements in environmental durability, including moisture and UV resistance, may be achieved through hydrophobic coatings, chemical crosslinking, UV-stabilizing additives, or interfacial modification strategies. Overall, by uniting the functionalities of water-activated and heat-softenable adhesives within one renewable material, this study defines a new class of cellulose-based adhesives that meet the growing demand for sustainable, high-performance alternatives to petroleum-derived systems.

## Materials and Methods

### Materials

Northern bleached softwood kraft pulp (NBSK) was kindly provided by Pulp and Paper Center (PPC; University of British Columbia, Canada). Sodium metaperiodate (NaIO_4_, 98%, Alfa Aesar), sodium chloride (NaCl, 98%, Alfa Aesar), sodium hydroxide (NaOH, 98%, Thermo Scientific), and hydroxylamine hydrochloride (NH_2_OH·HCl, 99%, Alfa Aesar) were purchased from Fisher Scientific, USA. Sodium borohydride (NaBH_4_ 98+%) was purchased from Acros Organics, USA. All reagents are used as received without further purification. Deionized water was used throughout the experiments.

### Fabrication of all-cellulose film adhesive

Sequential sodium metaperiodate oxidization and sodium borohydride reduction was employed to convert NBSK to dialcohol celluloses with different oxidation degrees. Firstly, 7 g of NBSK pulp was fully dispersed in 700 ml of water for 24 h, and then NaIO_4_ (1:1, 0.75:1, and 0.5:1, molar ratio to AGU) and 27.09 g of NaCl were added into the suspension and reacted in the dark for 72 h at room temperature. The obtained suspension was dialyzed against water for 1 week (dialysis tubing: 12 to 14 kDa, Spectra/Por molecularporous). After dialysis, 3.5 g of NaBH_4_ was added into the suspension reacted for 24 h, and then dialyzed against water. The dialcohol cellulose suspension after dialysis was dried in a homemade mold (width: 30 cm, length: 30 cm) at 55% relative humidity and 25 °C for 72 h, forming an all-cellulose film adhesive. For the oven drying process, the suspension was dried at 80 °C for 24 h.

### Determination of aldehyde content

The aldehyde content after the periodate oxidation was determined by the hydroxylamine hydrochloride titration method. Namely, 10 ml of 5 wt % NH_2_OH·HCl solution was adjusted to pH 3 and then mixed with 20 ml of periodate oxidized cellulose suspension (~0.1 g of solid content) under magnetic stirring for 24 h. The suspension was then titrated using 0.05 M NaOH to pH 3. The aldehyde content (mmol/g) was determined using Eq. (1):

$$\text{Aldehyde content}=\frac{V_{\text{NaOH}}\times C_{\text{NaOH}}}{m_{\text{cellulose}}}$$ (1)

where *V*_NaOH_ and *C*_NaOH_ are the volume and concentration of the NaOH solution, and *m*_cellulose_ is the dry cellulose weight in the suspension.

### Characterizations

ATR-FTIR spectra were obtained by INVENIO S (Bruker Optics Pty. Ltd., Billerica, MA, USA) with a diamond crystal at a resolution of 4 cm^−1^ in the range of 4,000 to 400 cm^−1^ for 1:1, 0.75:1, and 0.5:1 films, respectively. XRD diffractogram of 1:1, 0.75:1, and 0.5:1 films was obtained through a Bruker D8-Advance x-ray diffractometer (Bruker, Germany) equipped with cobalt radiation (40 kV and 50 mA) at a scanning speed of 1° min^−1^ from 2*θ* = 5° to 55°. DSC measurements were conducted using a TA Q1000 (TA Instruments, USA) under the following conditions: (a) the sample was initially cooled to 0 °C at a rate of 10 °C/min and held for 2 min; (b) it was then heated to 200 °C at the same rate; (c) finally, the sample was cooled back to 0 °C and reheated to 200 °C at a rate of 10 °C/min. UV–visible spectroscopy was used to measure the transmittance of the films in the range of 200 to 800 nm using a Lambda 35 (PerkinElmer, USA) at a scan rate of 30 nm min^−1^. Mechanical tests of all films were conducted using a uniaxial material testing system (Instron 5969, USA) at 25 °C and 55% relative humidity, with a 500-N load cell, a crosshead speed of 2 mm min^−1^, and a 10-mm fixture. Prior to testing, the samples were cut into rectangular shapes (30 mm in length, 3 mm in width, and approximately 0.04 mm in thickness). ^13^C CP-MAS NMR spectra of the original pulp and films with high-power proton decoupling were obtained using a 400-MHz Bruker ADVANCE solid-state widebore spectrometer. Samples were spinning at 6 kHz at magical angle. ^13^C pulse with a ramped amplitude at 50% was used for cross-polarization with a contact time of 5 ms. Relaxation delay was set to 2 s, and acquisition time was set to 50 ms. Data were processed with a 50-Hz line broadening exponential decay function prior to the Fourier transform. Before data acquisition, chemical shift values (ppm) were referenced with adamantane. All experiments were done at room temperature. Samples were ground into powder before tests. DMA of film (1:1) was performed on DMA-850 (TA Instruments, USA) with a temperature ramp from 30 to 120 °C at a heating rate of 3 °C/min.

### Morphology observation

The morphology of dialcohol cellulose was observed through a polarized optical microscope (POM) and TEM, while the morphology of the corresponding film was observed by SEM. Dialcohol cellulose (0.01 wt %) was deposited on glow-discharged formvar carbon copper grids, negatively stained with 2% uranyl acetate, and observed using Tecnai Spirit TEM under 120-kV accelerating voltage. OM and POM were conducted by POLYVAR (OPTI-TECH, Canada) using 1 wt % cellulose suspension. The microstructures and morphologies of dialcohol cellulose film were observed by Helios NanoLab 650 FIB-SEM at a working distance of 4 mm and an accelerating voltage of 5 kV. Before imaging, samples were sputter-coated with 10 nm of iridium (Leica EM MED020 Coating System, Leica Microsystems, Wetzlar, Germany). SAXS (XEUSS 2.0 instrument, Xenocs SAS, Grenoble, France) was employed to analyze the nanostructures of films. The x-ray wavelength *λ* was 1.54 Å. The sample-to-detector distance (SDD) was 1,143 mm, calibrated using silver behenate standard. 2D scattering patterns were captured by a Pilatus 300k detector (Dectris, Switzerland). Conversion of 2D data to 1D profiles was carried out using the Nika IGOR macro developed by Ilavsky [42]. All data were subtracted from the background. For SAXS analysis, the 2D scattering patterns were azimuthally integrated to obtain 1D intensity profiles, *I*(*q*). To better reveal the characteristic correlation distance, the profiles were Lorentz-corrected according to *I*_L(*q*)_ = *I*(*q*)*q^2^*, where *q* is the scattering vector. The characteristic internal spacing, *d*, was calculated from the peak position, *q*_max_, using *d* = 2π/*q*_max_.

### Adhesion properties

The adhesion strength was measured through a lap-shear test via a material testing system (Instron 5969, USA). Different substrates (basswood, copper, plastic, aluminum, and glass) were firstly prepared into rectangular slides (width: ~2 cm, length: ~8 cm), and a piece of dialcohol celluloses film (width: ~2 cm, length: ~2 cm, ~20 mg) was placed on one side of the substrates. After dropping ~1 ml of water on the film, the adhesion area was covered by another side of the substrates (total film mass: ~25 mg; total bonding area, *S*: ~4 cm^2^). After curing (1, 3, 5, and 7 d) under room temperature, the sample was tested on Instron 5969 with a 2,000-N load cell and crosshead speed of 2 mm/min. The adhesion strength was calculated by *F*/*S*, where *F* is the maximum force during lap shear and *S* is the bonding area. For heat activation, the 1:1 film (width: ~2 cm, length: ~2 cm, ~24 mg) was sandwiched between 2 pieces of wood substrates (width: ~2 cm, length: ~8 cm, thickness: ~2 mm) and hot pressed for different times (from 0.25 to 2 h) under different temperatures (from 70 to 200 °C). The adhesion strength after heat activation was tested through the same lap-shear tests. The shear block adhesion strength was measured on an MTS Flextest GT structural test machine with a loading rate of 5 mm/min. Wood blocks of 50.8 × 19 × 44.4 mm^3^ were adhered by 1:1 film adhesives under water activation and curing for 3 d. A small hole is drilled in the center for each sample to install a thermocouple and monitor the glue line temperature during mechanical testing. For testing under different temperatures, each sample was preheated in an oven for around 90 min until the glue line temperature reached the target temperature. Commercial PVA wood adhesive (LePage Multi-Purpose White Glue, Henkel Canada Corporation, Canada) was used as received for comparison. The lap shear samples were prepared using the same basswood substrate geometry and bonding area as the cellulose film adhesive, and cured under the same ambient conditions before testing

## Data Availability

All data supporting the findings of this study are available within the paper and its Supplementary Materials. In addition, the datasets generated or analyzed during this study are available from the corresponding author on reasonable request.
