# Supplementary material for: Water–Heat Dual-Activated Scalable Cellulose Film Adhesive
Source: Research (Wash D C). 2026 Aug 5;9:1393. doi: 10.34133/research.1393 (PMC13445968; doi:10.34133/research.1393)
Supplement: Supplementary file 1 [file research.1393.f1.docx]

Supplementary Information for

**Water-Heat Dual-Activated Scalable Cellulose Film Adhesive**

Supporting Information Text

**Discussion for Fig. S9-S10.** Additionally, the morphologies of the film were further examined by SEM at both surface (**Fig. 3f and** **Fig. S9a-c**) and cross-section (**Fig. S10a-c**). At low oxidation degrees, the film was mainly composed of long microfibers. After increasing the oxidation degree, the film surface became smoother and decorated with short microfibers. Further increasing the oxidation degree leads to the formation of a smooth film without any microfibers. The cross-section of films also revealed similar trends: the 0.5:1 film exhibited more cracks, while the 1:1 film demonstrated a more compact structure. Macroscopically, these interconnected microfibers contributed to high tensile strength, whereas the presence of fragments and cracks within the film at lower oxidation degrees induced stress concentration during deformation, resulting in lower fracture strain.

**Discussions for Fig. S18-S21.** Discussion: Notably, the nano-scaled structures and increased chain mobility of dialcohol cellulose allow them to penetrate substrates more easily compared to cellulose microfibers or fragments, resulting in superior adhesion strength. This was indicated by the wood failure of 1:1 film after tests as shown in Fig. S18-19; whereas the 0.5:1 and 0.75:1 film primarily exhibited cohesive failure, indicating insufficient mechanical interlocking. Similarly, long curing time also ensures sufficient mechanical interlocking, as reflected in Fig. S20-S21, where all samples demonstrated typical wood failure, with more severe failure observed in samples cured for 3, 5, and 7 days.

**Fig. S1.** Schematic illustration of sequential oxidation and reduction

**Fig. S2.** Force-displacement curve of commercial polyvinyl acetate (PVA) wood adhesive (LePage® Multi-Purpose White Glue, Henkel Canada Corporation, Canada) and the corresponding lap shear strength.

**Fig. S3.** Optical images and polarized optical images of 0.5:1 group.

**Fig. S4**. Optical images and polarized optical images of 0.75:1 group.

**Fig. S5**. Optical images and polarized optical images of 1:1 group.

**Fig. S6**. Optical photos of dialcohol celluloses suspension with different oxidation degrees.

**Fig. S7**. TEM images of dialcohol nanocelluloses under different oxidation degrees.

**Fig. S8**. Yield of dialcohol celluloses of different oxidation degrees.

**Fig. S9**. Surface morphologies of all-cellulose films with different oxidation degrees.

**Fig. S10**. Cross-section morphologies of all-cellulose films with different oxidation degrees.

**Fig. S11**. Digital photos of all-cellulose films with different oxidation degrees.

**Fig. S12**. UV-Vis curves of all-cellulose films with different oxidation degrees.

**Fig. S13**. (a) Toughness, (b) elastic modulus and (c) tensile stress of all-cellulose films with different oxidation degrees.

**Fig. S14**. Schematic illustration of wood sample for lap shear tests.

**Fig. S15**. The solid content of adhesive samples of (a) different oxidation degrees, and (b) different curing time.

**Fig. S16**. Force-displacement curves of all-cellulose film adhesives (1:1 group) with different curing time.

**Fig. S17**. A piece of wood bonded by film adhesive (1:1 group) holding an adult (75 kg).

**Fig. S18**. Digital photos of wood adhesive samples after lap shear tests for different oxidation degrees. Curing time: 3 days.

**Fig. S19.** Microscopy images of wood surface after lap shear tests for different oxidation degrees. Curing time: 3 days.

**Fig. S20.** Digital photos of wood adhesive samples (1:1 group) after lap shear tests for different curing time.

**Fig. S21**. Microscopy images of the wood surface after lap shear tests (1:1 group) for different curing times.

**Fig. S22**. Force-displacement curves for shear block tests of film adhesive (1:1 group) under different temperatures.

**Fig. S23**. (a) TGA and (b) corresponding DTG curves of pulp and 1:1 dialcohol cellulose. (c) ATR-FRIR spectra before and after placing the film under 200 °C for 30 mins.

**Fig. S24**. Schematic illustration of adhesion mechanisms under heat or water activation.

**Fig. S25**. Force-displacement curves of 1:1 film adhesive on a paper bag and corresponding photo showing a typical cohesive failure.

**Fig. S26**. Force-displacement curves of different substrates bonded by 1:1 cellulose film adhesive and (b) corresponding lap shear strength.

**Fig. S27**. Long-term storage stability of film adhesive. (a) ATR-FTIR spectra of original 1:1 film and film after 1-year storage. (b) Force-displacement curves of original 1:1 film and film after 1-year storage, and (c) corresponding lap shear strength (condition: heat-activated under 100 °C, 0.25 h).

**Table S1**. Comparison of lap shear strength with previously reported wood adhesives

| Main component | Activation | Lap shear strength / MPa | Ref. |
| --- | --- | --- | --- |
| Dialcohol cellulose | Heat | 2.23 ± 0.16 | This work |
| Dialcohol cellulose | Water | 3.08 ± 0.14 | This work |
| Uncondensed lignins | Heat | 1.28 ±  0.17 | 5 |
| Soy protein-based | Heat | 2.08 | 36 |
| Dialdehyde cellulose | Heat | 2.71 | 37 |
| Soy protein | Heat | 1.96 | 39 |
| Tosyl cellulose | Heat | 2.31 | 40 |
| Soy protein | Heat | 2.23 ± 0.06 | 41 |
| Starch | Heat | 3.21 | 42 |

**Table S2**. Comparison of lap shear strength towards various substrates bonded with 1:1 cellulose film adhesive

| Substrates | Activation | Lap shear strength / MPa |
| --- | --- | --- |
| Bass wood | Heat | 2.23 ± 0.16 |
| Bass wood | Water | 3.08 ± 0.14 |
| Glass | Water | 0.71 ±0.36 |
| Copper | Water | 0.23 ± 0.03 |
| Aluminum | Water | 0.28 ± 0.04 |
| PET | Water | 0.31 ± 0.11 |
